# Inhibition of glucose-6-phosphate dehydrogenase sensitizes cisplatin-resistant cells to death

**DOI:** 10.18632/oncotarget.4945

**Published:** 2015-08-17

**Authors:** Daniela Catanzaro, Edoardo Gaude, Genny Orso, Carla Giordano, Giulia Guzzo, Andrea Rasola, Eugenio Ragazzi, Laura Caparrotta, Christian Frezza, Monica Montopoli

**Affiliations:** ^1^ Department of Pharmaceutical and Pharmacological Sciences, University of Padova, Padova, Italy; ^2^ MRC Cancer Unit, University of Cambridge, Hutchison/MRC Research Centre, Cambridge Biomedical Campus, Cambridge, UK; ^3^ IRCCS “E. Medea”, Conegliano, Italy; ^4^ Department of Radiological, Oncological and Pathological Sciences, Sapienza University of Rome, Roma, Italy; ^5^ Department of Biomedical Sciences, University of Padova, Padova, Italy

**Keywords:** cisplatin, drug resistance, cancer metabolism, PPP, transmitochondrial hybrids

## Abstract

The mechanisms of cisplatin resistance, one of the major limitations of current chemotherapy, has only partially been described. We previously demonstrated that cisplatin-resistant ovarian cancer cells (C13), are characterized by reduced mitochondrial activity and higher glucose-dependency when compared to the cisplatin-sensitive counterpart (2008). In this work we further characterized the role of metabolic transformation in cisplatin resistance. By using transmitochondrial hybrids we show that metabolic reprogramming of cisplatin-resistant cell is not caused by inherent mtDNA mutations. We also found that C13 cells not only present an increased glucose-uptake and consumption, but also exhibit increased expression and enzymatic activity of the Pentose Phosphate pathway (PPP) enzyme Glucose-6-Phosphate Dehydrogenase (G6PDH). Moreover, we show that cisplatin-resistant cells are more sensitive to G6PDH inhibition. Even if the metabolomic fingerprint of ovarian cancer cells remains to be further elucidated, these findings indicate that PPP offers innovative potential targets to overcome cisplatin resistance.

## INTRODUCTION

Cisplatin is one of the most potent anticancer agents used in the treatment of various solid tumors including testicular, lung and ovarian cancer [1]. Despite the chemotherapeutic advances of the last decades, cisplatin still remains the first-line chemotherapeutic agent against epithelial malignancies, used alone or in combination with radiotherapy and/or other anticancer compounds. Cisplatin has been shown to induce the formation of inter- and intra-strand nuclear DNA (nDNA) cross-links that, by hindering both RNA transcription and DNA replication, lead to cell cycle arrest and apoptosis [2]. Cisplatin has also been shown to bind to mitochondrial DNA (mtDNA), which, unlike nDNA, is more susceptible to the onset of mutations that lead to mitochondrial dysfunction, oxidative stress and mitochondria-dependent apoptosis [3, 4, 5]. The cisplatin therapeutic effectiveness is limited by side effects (ototoxicity, nephrotoxicity and neurotoxicity) [6] and by the emergence of resistance [7], a multi-factorial phenomenon, linked to reduced drug accumulation, inactivation by thiol-containing species, increased repair of platinum-DNA adducts, enhanced tolerance to cisplatin adducts and desensitization to cell death pathways [8, 9, 10]. These mechanisms are cell line-dependent, so that a particular tumor may exhibit one, two or even all the above-mentioned mechanisms [11].

In our previous studies we demonstrated that cisplatin-resistant ovarian cancer cells exhibit lower levels of Reactive Oxygen Species (ROS) and increased steady state levels of reduced glutathione (GSH) [12] suggesting that cisplatin-resistance can also be associated with the variation of intracellular redox status. It is well established that GSH plays a key role in the antioxidant capabilities of cancer cells, but it is important to remember that GSH also acts forming complexes with cisplatin, thereby reducing the amount of intracellular drug available for interaction with all nucleophilic sites [13].

Cancer cells undergo profound metabolic changes required to fuel the biosynthetic demands of cell growth and division [14, 15] and to maintain the redox homeostasis. This metabolic rewiring is orchestrated by oncogenes and tumor suppressors, which fine tune several metabolic pathways, including glycolysis, oxidative phosphorylation, the pentose phosphate pathway and glutaminolysis [16]. Indeed, the increase in glucose metabolism, that hallmarks most cancer cells, is instrumental not only for ATP generation or for the synthesis of nucleotides and amino acids, but also for the maintenance of redox homeostasis via the NADPH-producing Pentose Phosphate Pathway (PPP) [17]. The rate-limiting and “gatekeeper” enzyme of the PPP is glucose-6-phosphate dehydrogenase (G6PDH), whose activity is regulated by the availability of its substrate and by the NADP+/NADPH ratio [18] and directly reflects the partitioning of glucose utilization between glycolysis and oxidative PPP [19]. However, whether the metabolic reprogramming of cancer supports cisplatin resistance is currently unknown.

To shed some light on the metabolic determinants of cisplatin resistance, we had investigated the metabolic changes that occur in cisplatin–resistant ovarian cancer cells (C13), finding that they exhibit reduced oxygen consumption and increased dependency on glucose, when compared to their cisplatin-sensitive counterpart (2008) [20]. In this work we have further characterized the role of metabolic transformation in cisplatin resistance. We show that cisplatin induces deregulation of mitochondrial function in resistant cells, in accord with their increased usage of glucose, and that cisplatin-resistant cells rely on the PPP to overcome cisplatin cytotoxicity. We also demonstrate that the inhibition of the limiting enzyme of the PPP, G6PDH, restores cisplatin sensitivity in cisplatin-resistant cells.

## RESULTS

### Role of mtDNA polymorphisms in cisplatin resistance

**Figure 1:**
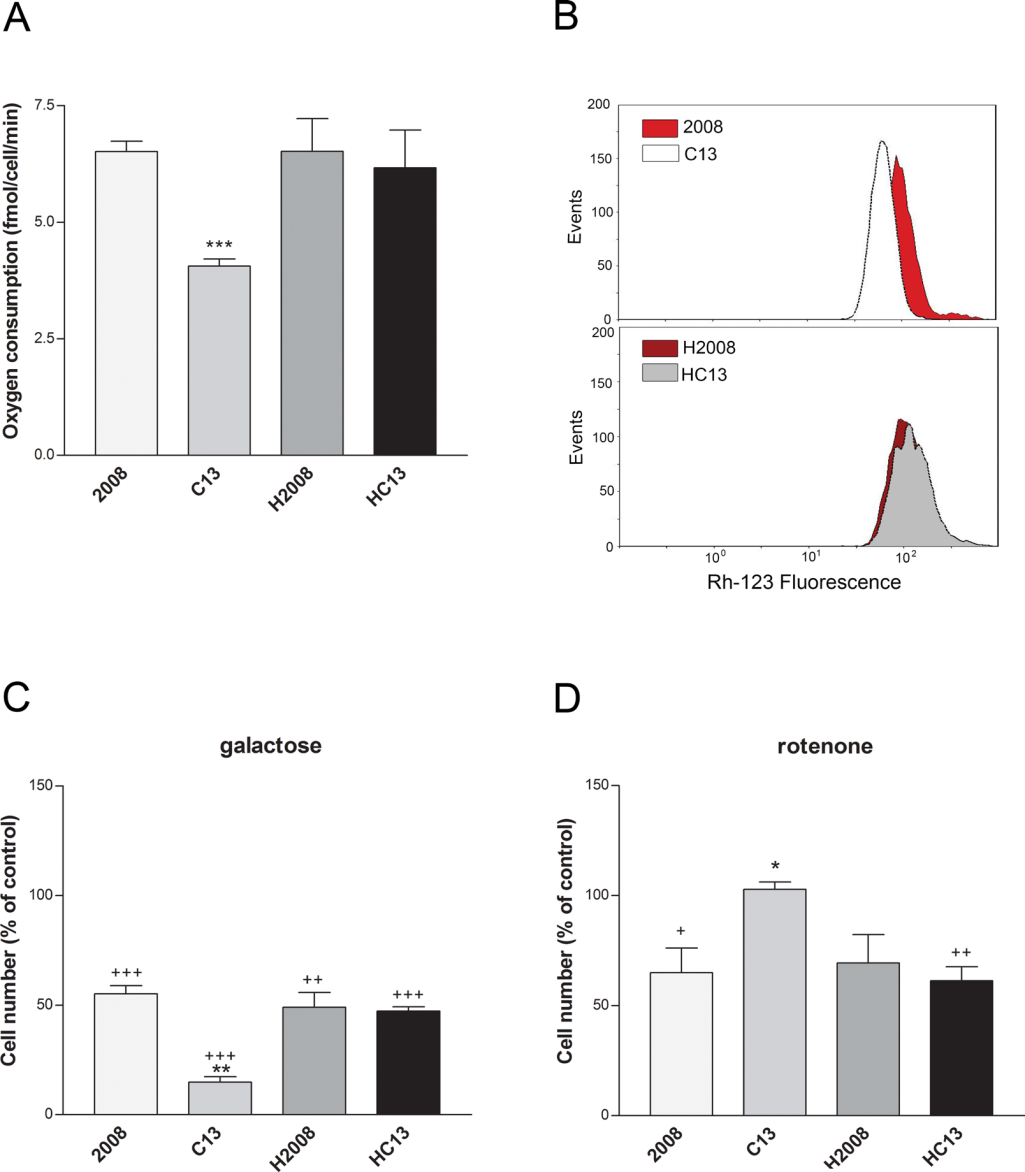
Mitochondrial function in cancer cisplatin-resistant cells and their derived cybrids **A.** Oxygen consumption of ovarian cancer cells (2008-C13) and their derived cybrids (H2008-HC13). **B.** Representative plots of the mitochondrial potential (ΔΨm) measured by flow cytometry. **C.** Effect of 5 mM galactose and **D.** 0.1 μM rotenone on cell viability after 24 hours of treatment. Data are expressed as percentage of cell number compared to the relative control. The data were obtained from at least 3–5 independent cultures. ***p < 0.001, **p < 0.01, *p < 0.05; C13 *vs* 2008. +++*p* < 0.001, ++*p* < 0.01, +*p* < 0.05; treatment *vs* control.

We initially investigated whether the mitochondrial dysfunction observed in cisplatin-resistant cells is a consequence of the damaging effects of cisplatin on mtDNA. To this aim, the mtDNA of C13 cells and of their cisplatin-sensitive counterpart 2008, was sequenced (Table 1). These analyses revealed three major polymorphisms in C13 cells: 8156 G>T, 12018 C>T, 13828 C>T. Their putative pathogenicity was assessed by PolyPhen (http://genetics.bwh.harvard.edu/pph2/), a software application that predicts the impact of an amino acid substitution on the structure and function of a protein. The predicted pathogenicity of the 8156 G>T and 13828 C>T polymorphisms was modest, whereas the 12018 C>T polymorphism resulted mildly pathogenic. In order to investigate if these mtDNA polymorphisms contributed to the altered mitochondrial function and cisplatin resistance, we generated trans-mitochondrial cytoplasmic hybrids (cybrids) (Supplementary Figure 1A for a schematic representation of the experiment). Interestingly, the cybrid lines (H2008 and HC13) were equally sensitive to cisplatin, as demonstrated by trypan blue exclusion assay and by Annexin V assay (Supplementary Figure 1B–1C). In line with our previous observations, cisplatin-resistant C13 cells presented lower oxygen consumption (Figure 1A) and lower mitochondrial potential (Figure 1B) when compared to 2008 cells. Interestingly, both respiration and mitochondrial potential defects were restored in the cybrid cell line HC13 and were comparable to the original 2008 cells (Figure 1A–1B). We further assessed the presence of mitochondrial dysfunction in C13 cells and in cybrid cell lines by growing cells in galactose medium. Of note, C13 cells were extremely sensitive to these culture conditions (Figure 1C), whereas their viability was not affected by long-term incubation with the Complex I inhibitor rotenone (Figure 1D). In further support of the restoration of mitochondrial function in cybrids, growth of HC13 cells was not affected by galactose, and HC13 cells became sensitive to rotenone (Figure 1C–1D). Together, these results suggest that the mitochondrial polymorphisms found in C13 cells do not directly contribute to the observed mitochondrial dysfunction and do not play a role in cisplatin resistance.

**Table 1:** mtDNA sequence of human ovarian cancer cells

2008		
POLYMORPHISM	GENE	VARIATION
66delG	MT-DLOOP	
309insC	MT-DLOOP	
315insC	MT-DLOOP	
379 A>T		
709 G>A	MT-RNR1	non cod
1438 A >G	MT-RNR1	non cod
1888 G > A	MT-RNR2	non cod
4769 A > G	MT-ND2	syn
6734 G > A	MT-CO1	syn
8860 A > G	MT-ATP6	T-A
15287 T > C	MT-CYB	F-L
15326 A > G	MT-CYB	T-A
16519 T > C	MT-DLOOP	

C13		
POLYMORPHISM	GENE	VARIATION
66delG	MT-DLOOP	
263 A > G	MT-DLOOP	
315insC	MT-DLOOP	
379 A > T		
709 G > A	MT-RNR1	non cod
1438 A > G	MT-RNR1	non cod
1888 G > A	MT-RNR2	non cod
4769 A > G	MT-ND2	syn
6734 G > A	MT-CO1	syn
**8156 heteroplasmy** G > T*	**MT-CO2**	**V-L** **LD = 0,53**
8860 A > G	MT-ATP6	T-A
**12018 heteroplasmy** C > T**	**MT-ND4**	**T-I** **LD = 0,69**
**13828 heteroplasmy** C > T**	**MT-ND5**	**L-P** **LD = 0,05**
14470 T > A	MT-ND6	syn
15287 T > C	MT-CYB	F-L
15326 A > G	MT-CYB	T-A
16519 T > C	MT-DLOOP	

* 30% of mtDNA polymorphism rate in C13compared to 2008 cells.

** 50% of mtDNA polymorphism rate in C13 compared to 2008 cells.

### Cisplatin-resistant cells exhibit defects in mitochondrial morphology and a reduction in mitochondrial mass

**Figure 2:**
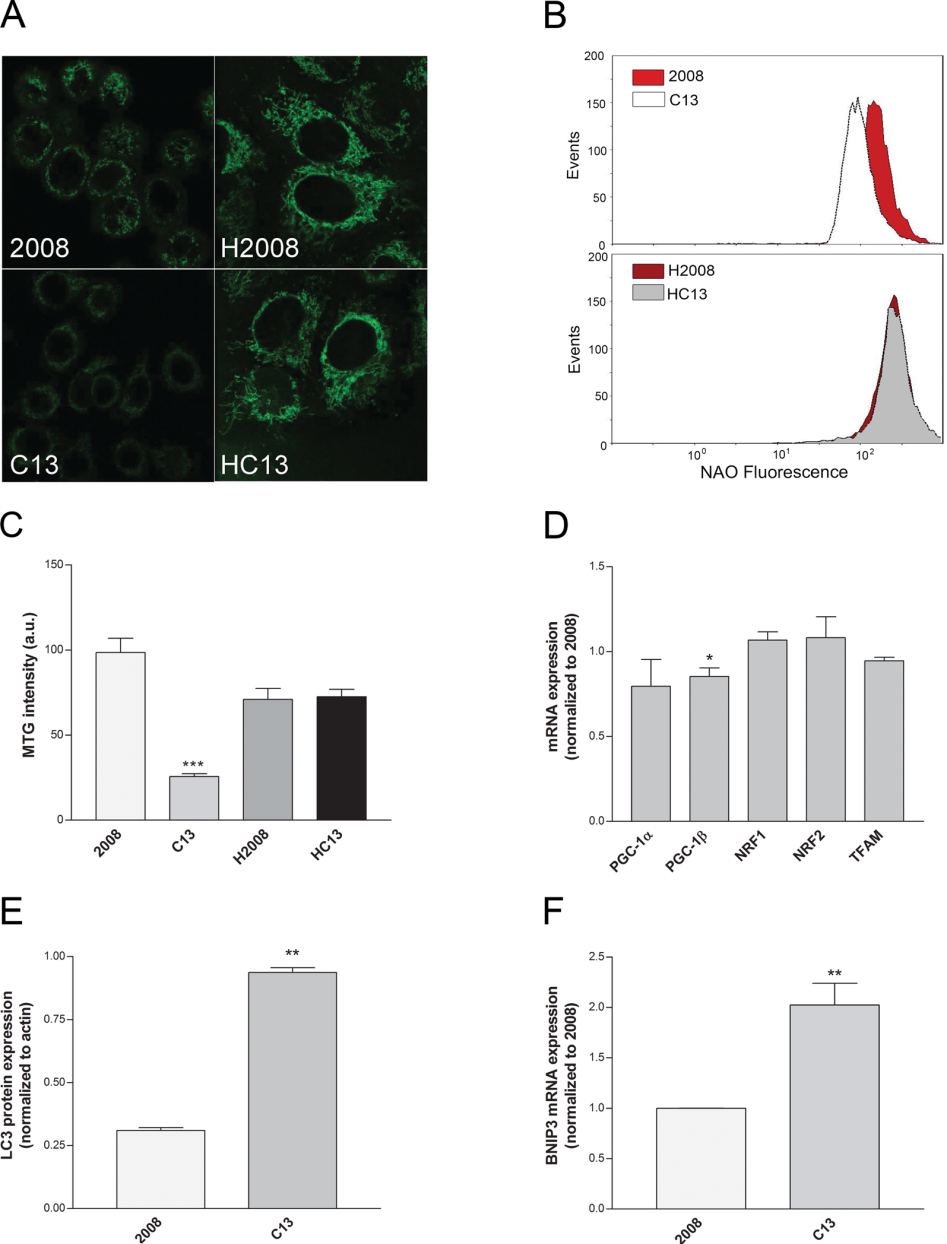
Cisplatin-resistant cells present a reduced mitochondrial mass **A.** Representative confocal images of Mitotracker Green (MTG) staining. **B.** Representative plots of Acridine Orange 10-Nonyl bromide (NAO) mean fluorescence intensity measured by flow cytometry.**C.** MTG mean fluorescence intensity, calculated after a volumetric reconstruction of the mitochondrial network. **D.** Expression levels of genes involved in mitochondrial biogenesis or **F.** in mitophagy as tested by qRT-PCR. All genes were normalized to β-actin as endogenous control. **E.** LC3 protein expression measured from western blotting. The data were obtained from at least 3–5 independent cultures. ***p < 0.001, **p < 0.01, *p < 0.05; C13 *vs* 2008.

We wanted to investigate whether decreased oxygen consumption observed in C13 cells was caused by a decrease in mitochondrial mass. To this aim, cells were stained with mitotracker green (MTG), a potential-independent mitochondrial probe, and mitochondrial mass was analysed using confocal imaging. Of note, while the mitochondrial network of C13 cells appeared scattered and less structured than in 2008 cells (Figure 2A, left panels), no differences in mitochondrial morphology between H2008 and HC13 were observed (Figure 2A, right panels). Importantly, C13 cells showed a dramatic decrease in mitochondrial mass measured by Nonyl-Acridine Orange (Figure 2B), which was consistent with a decrease in MTG staining with respect to their 2008 counterpart (Figure 2C). Since the expression of genes involved in mitochondrial biogenesis, except for PGC-1β, was not significantly different between C13 and 2008 (Figure 2D), we reasoned that the striking reduction in mitochondrial mass could be caused by induction of mitochondrial specific autophagy (mitophagy). Indeed, C13 cells exhibited a marked increase in LC3 staining (Figure 2E), associated to a significant overexpression of BNIP3 (Figure 2F), two key markers of activated autophagy. These results indicate that cisplatin-resistant cells exhibit defects in mitochondrial morphology and a reduction in mitochondrial mass, which are potentially associated to an increased activation of mitophagy.

### Deregulation of glucose and glutamine metabolism in cisplatin-resistant cells

**Figure 3:**
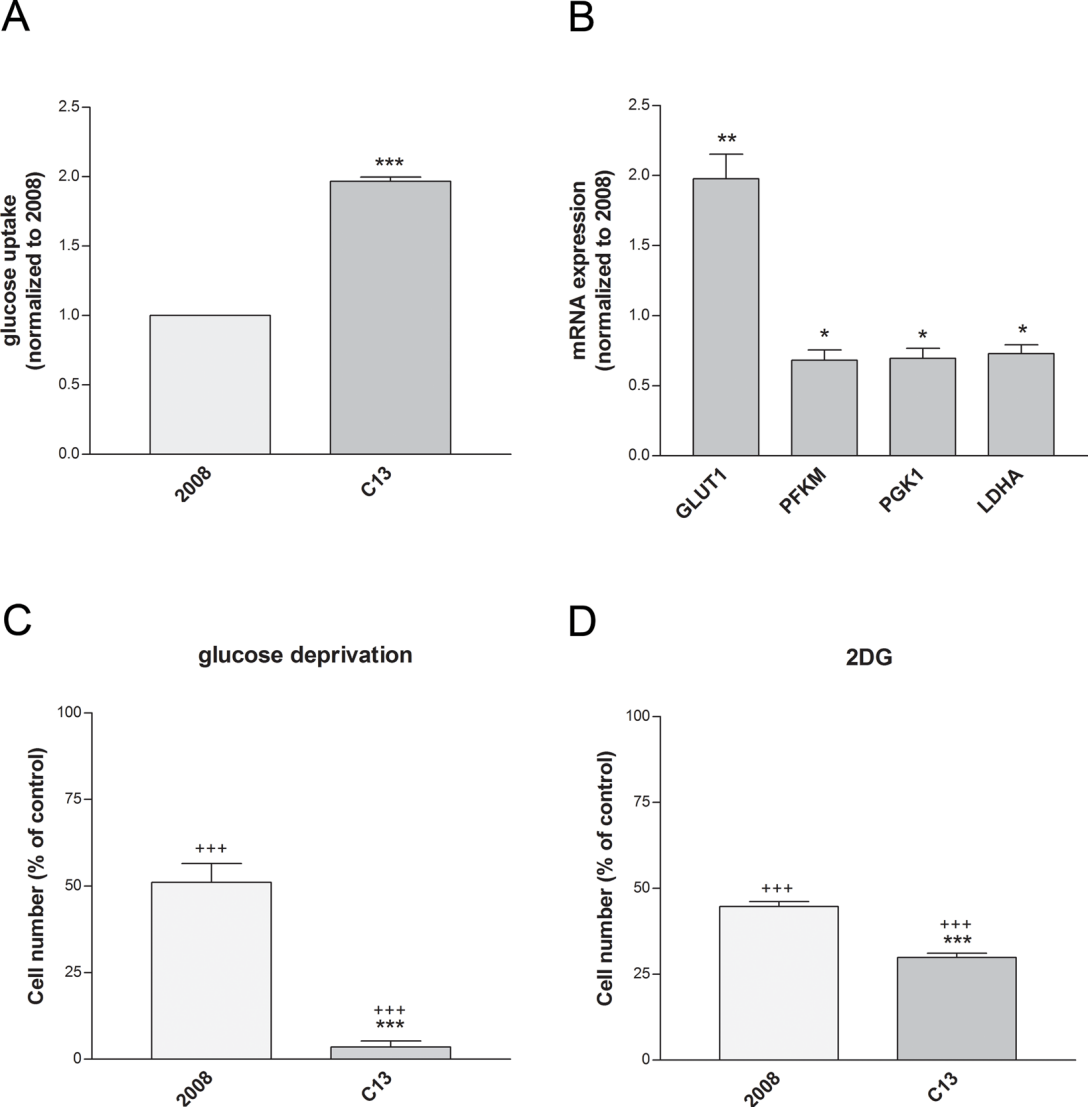
Cisplatin-resistant cells show an increased dependency to glucose **A.** Glucose uptake measured after incubation with the glucose analogue 6-NBDG. Data are normalized to cisplatin-sensitivecells. **B.** Expression levels of glycolytic genes measured by qRT-PCR. All genes were normalized to β-actin. **C–D.** Cell viability after 24 hours of glucose deprivation with (D) or without (C) 1 mM 2-DG. Data are expressed as percentage of cell number compared to control. The data were obtained from at least 3–4 independent cultures. ***p < 0.001, **p < 0.01, *p < 0.05; C13 *vs* 2008. +++*p* < 0.001; treatment *vs* control.

In line with a compensatory activation of glycolysis in the presence of mitochondrial defects, C13 cells exhibited increased glucose uptake (Figure 3A). Among the glycolytic enzymes investigated, only the glucose transporter GLUT1 was up-regulated in these cells (Figure 3B). Of note, cisplatin-resistant cells exhibited higher sensitivity to glucose deprivation (Figure 3C) and the incubation with the glycolysis inhibitor 2-Deoxyglucose (2DG) led to significant cell death of these cells (Figure 3D). To support these data we used also another cancer cell line (human cervix squamous cell line, A431) and its relative cisplatin resistant counterpart (A431pt). A431pt, similarly to C13, presented a higher glucose-dependency, increased GLUT1 expression, and major sensitivity to galactose medium (Supplementary Figure 2A–2D). Together, these results show that cisplatin-resistant cells increase their demands of glucose and are more sensitive to inhibition of glycolysis, when compared to the cisplatin-sensitive counterpart.

**Figure 4:**
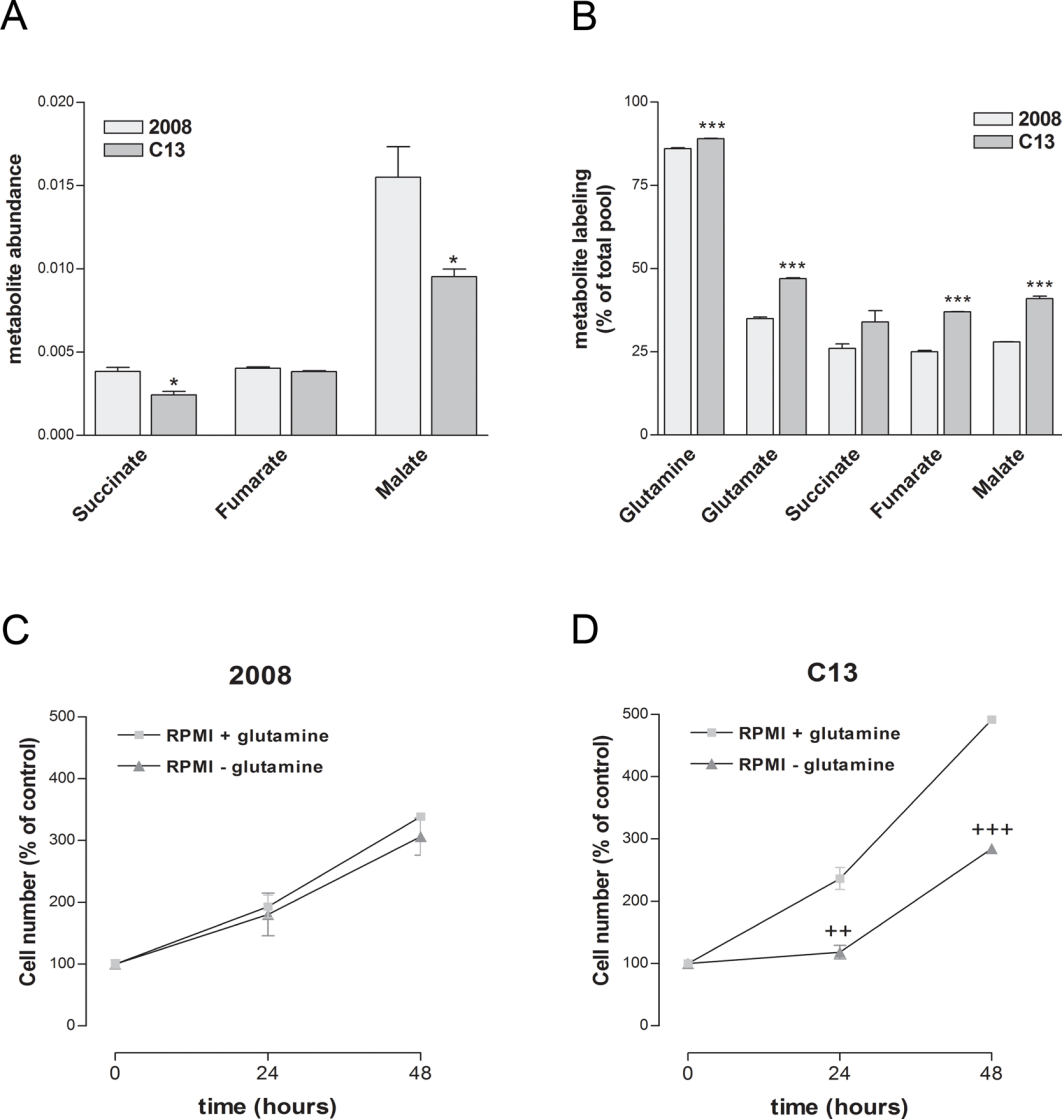
Cisplatin-resistant cells present an increased dependency on glutamine for TCA intermediates biosynthesis **A.** Abundance of TCA intermediates measured using LC-MS normalised to total ion current. **B.** Incorporation of 13C-labelled carbons into glutamate, succinate, fumarate and malate after growing cells for 24 hours in the presence of [U-^13^C]glutamine. **C–D.** Effect of glutamine deprivation on 2008 (C) and C13 (D) cell viability measured by trypan blue exclusion assay. The data were obtained from at least three independent cultures. ***p < 0.001, *p < 0.05; C13 *vs* 2008. +++*p* < 0.001, ++*p* < 0.01; treatment *vs* control.

Glutamine is a major source of carbons for the mitochondria of cancer cells [21, 22]. In order to investigate the biosynthetic role of glutamine, we incubated C13 and 2008 cells with uniformly labelled [U-^13^C]glutamine and analysed the isotopologue distribution of tricarboxylic acid (TCA) cycle intermediates. While the total pool of some TCA cycle intermediates, including succinate and malate, was lower in C13 than in 2008 cells (Figure 4A), the incorporation of glutamine-derived carbons into glutamate, succinate, fumarate and malate was significantly higher in cisplatin-resistant cells (Figure 4B). These results suggest that in the presence of deregulated mitochondrial function, glutamine becomes a privileged source of carbon for C13 cells. In line with a functional relevance of glutamine, C13 cells, differently from 2008 cells, showed a marked decrease in cell proliferation when cultured in glutamine-free media (Figure 4C and 4D).

**Figure 5:**
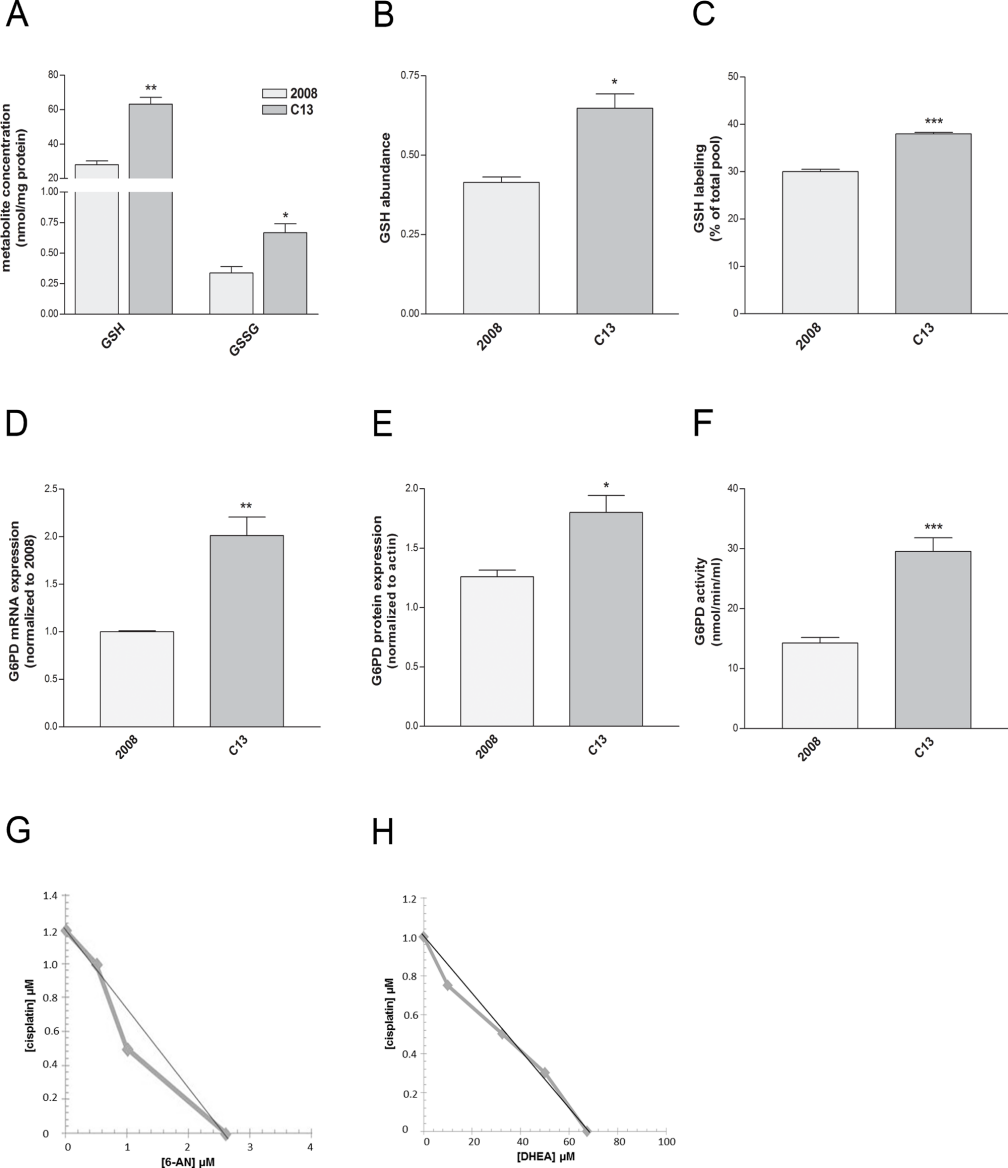
Redox homeostasis is required for survival of C13 cisplatin-resistant cells **A–B.** Cellular GSH and GSSG content measured by enzymatic assay (A) and GSH abundance (normalised to total ion count) measured by LC-MS (B). **C.** Intracellular levels of ^13^C_5_-GSH after growing cells for 24 hours in the presence of [U-^13^C]glutamine. Data are normalized on the total pool of intracellular GSH (B) **D.** G6PD mRNA levels quantified by qRT-PCR, **E.** protein expression measured by western blotting and **F.** G6PDH enzymatic activity of 2008 and C13 cells. **G–H.** Isobologram of cisplatin-resistant cells showing additive effect of 6-AN or DHEA in association with cisplatin treatment. Data are expressed as percentage of cell number compared to control. The graph was obtained using iso-effective drug concentrations causing 25% of cytotoxic effect. Straight line indicates the theoretical additivity line. The data were obtained from at least 3–5 independent cultures. ***p < 0.001, **p < 0.01, *p < 0.05; C13 *vs* 2008. +++*p* < 0.001; treatment *vs* control.

Besides being a carbon source for the TCA cycle, glutamine is a key precursor of glutamate, required, among many functions, for the biosynthesis of glutathione (GSH), a major redox buffer in the cells. GSH has been proposed as an important molecule to sustain cisplatin resistance, and the overexpression of enzymes involved in GSH biosynthesis has been documented in these cells [23]. Although GSH *vs* GSSG ratio was similar between C13 and 2008 cells (Supplementary Table 1), the amount of GSH and GSSG was significantly higher in C13 cells (Figure 5A–5B). Of note, the contribution of glutamine to GSH was increased in C13 cells (Figure 5C), suggesting that a portion of glutamine is used by cisplatin-resistant cells to sustain GSH biosynthesis. These results indicate that the metabolic reprogramming of cisplatin-resistant cells may contribute to redox buffering.

### Inhibition of Glucose-6-Phosphate Dehydrogenase (G6PDH) sensitizes cisplatin-resistant cells

Several lines of evidence suggest that, to maintain redox homeostasis, cancer cells engage into the PPP, a major source of NADPH for the cells. We hypothesized that, in addition to increasing GSH biosynthesis, C13 cells might exploit the PPP pathway to compensate for the increased oxidative stress. G6PDH is a major checkpoint for the activity of the PPP. Interestingly, G6PDH mRNA (Figure 5D), protein expression (Figure 5E) and activity (Figure 5F), were increased in C13 cells, when compared to 2008. To better understand the relevance of this metabolic pathway in cisplatin resistance, cells were incubated with a combination of 6-aminonicotinamide (6-AN, competitive G6PDH inhibitor [24]) or dehydroepiandrosterone (DHEA, uncompetitive G6PDH inhibitor [25]) and cisplatin at concentrations lower than IC_50_. In order to appropriately assess the effects of the combination of 6-AN or DHEA and cisplatin, we performed isobolographic analysis. The linearity of the iso-effective concentrations producing 25% of cytotoxic effect strongly indicates additivity (Figure 5G–5H). On the contrary, the cytotoxic effect on 2008 was only due to the prevalent cisplatin activity, which hindered the overall effect, so an isobolograph could not be generated. Of note also in A431pt G6PDH expression and mRNA were increased when compared to A431 (Supplementary Figure 3A–3B) similarly to C13. By performing isobolographic analysis on iso-effective concentrations of cisplatin and 6-AN or DHEA producing 25% of cytotoxic effect, in A431pt cells, the observed curves deviate from the theoretical line show additivity for 6-AN and a modest synergism for DHEA (Supplementary Figure 3C–3D). Taken together, these results suggest that cisplatin-resistant cells exploit the oxidative PPP as a resistance mechanism. Both resistant cell lines did not show cross-resistance with other chemotherapeutic agents, including paclitaxel and doxorubicin (see Supplementary Figure 4).

## DISCUSSION

Cisplatin cytotoxicity has been originally ascribed to interactions between cisplatin and nuclear DNA, which lead to the formation of adducts that activate the apoptotic machinery [2]. More recent data suggest that only 5–10% of cisplatin is bound to DNA, whilst other nucleophilic molecules such as phospholipids, cytoskeletal and membrane proteins, and mtDNA are targeted by the drug, suggesting that other mechanisms may explain cisplatin toxicity [26, 27]. Unfortunately, the therapeutic effectiveness of cisplatin is limited by the onset of cisplatin resistance [7], whose mechanisms are still not fully understood. Emerging evidence supports the idea that the deregulated cell metabolism could sustain drug resistance [28]. In this work we have investigated the contribution of metabolic reprogramming to cisplatin resistance and revealed novel metabolic liabilities of cisplatin-resistant cells.

Our previous research showed that cisplatin-resistant ovarian cancer cells (C13) present defective mitochondrial bioenergetics [12, 20]. By generating transmitochondrial hybrids [29, 30, 31] we demonstrate here that the mitochondrial polymorphisms found in C13 cells are not causative of the different mitochondrial asset previously observed. We also show that C13 mitochondrial dysfunction is not due to inherent respiratory chain defects but, rather, by a decrease in mitochondrial biomass. The increased mitochondrial turnover may be required to clear dysfunctional mitochondria that accumulate in cancer cells, and to maintain mitochondrial metabolism, as recently proposed by Strohecker *et al*. [32]. In line with these findings, we observed that the metabolic activity of C13 cells measured by incubating cells with [U-^13^C]glutamine is even higher than the cisplatin-sensitive counterpart. Therefore, our results suggest that a high mitochondrial turnover could compensate for inherent mitochondrial defects in cisplatin-resistant cells.

We then analyzed other aspects of the metabolic reprogramming of cisplatin-resistant cells, including glycolysis, glutaminolysis, and metabolic pathways involved in redox homeostasis. Cisplatin-resistant cells exhibited increased glucose uptake, overexpression of the glucose transporter GLUT1, and increased glutaminolysis, which make these cells more sensitive to glucose and glutamine deprivation. It is worth noting that glucose and glutamine are not mere carbon sources but they also contribute to the redox homeostasis of these cells. The activation of antioxidant pathways is now emerging as a crucial adaptive mechanism involved in drug resistance and a role for increased GSH biosynthesis has been proposed to provide resistance to cisplatin [23, 33, 34]. Consistent with this view, we found that glutamine-derived glutamate is utilized for GSH biosynthesis in cisplatin-resistant cells. In support of a metabolic rewiring of glucose towards antioxidant pathways, we found that the expression and enzymatic activity of G6PDH, a key enzyme of the PPP, were elevated in resistant cells as compared to sensitive cells. Moreover, the combined treatment with the G6PDH inhibitors 6-AN or DHEA and cisplatin, showed a selective additive effect on cisplatin-resistant cells, suggesting that upregulation of G6PDH activity could be a targetable mechanism of chemotherapeutic resistance to cisplatin. In leukemia cells, it has been documented that the acquisition of daunorubicin resistance is accompanied by elevation of the oxidative PPP. But adriamycin/doxorubicin-resistant MCF-7 cells display reduced G6PDH and PPP activity compared with sensitive cells, suggesting that a hyperactive PPP sensitizes cells to anthracyclines [17]. Differently from the contradictory results obtained with anthracyclines, our data clearly indicate that the PPP inhibition may be peculiar for cisplatin resistance, offering a novel synthetic lethality approach. These results are in line with the concept of induced essentiality, where by cancer cells become sensitive to the inhibition of the very same metabolic pathways that evolved as mechanisms of resistance from chemotherapeutic agents [35, 36].

Taken together our results show that profound metabolic changes underpin cisplatin resistance. Besides reduction of mitochondrial mass and overall mitochondrial function, these cells increase their demand of glucose and glutamine, which are in part used to maintain the cell's antioxidant defenses. Chemotherapy designed to target metabolic pathways is a new approach, and potentially more effective to inhibit tumor cell growth [37]. Our work demonstrated that the combination of cisplatin treatment with inhibition of the PPP enzyme G6PDH, can remarkably improve the cytotoxic effects of cisplatin and can help to overcome cancer resistance to cisplatin treatment.

## MATERIALS AND METHODS

### Cell lines

**Human ovarian carcinoma cell lines** (2008 wild type and C13 cisplatin-resistant cells) were grown in Roswell Park Memorial Institute medium (RPMI 1640) supplemented with 10% fetal bovine serum (FBS), 4 mM glutamine, 100 U/ml penicillin and 100 μg/ml streptomycin, in humidified condition at 5% CO_2_ and 37°C.

**206-ρ° cells** derived from mtDNA depletion of 143B-TK^−^ osteosarcoma cells (kind gift of Andrea Martinuzzi, IRCSS E. Medea-La Nostra Famiglia association) were cultured as previously described [38].

**Transmitochondrial cybrid cell lines** (H2008 and HC13) were generated by polyethylene glycol fusion of enucleated 2008 and C13 with the mtDNA-less (ρ^0^) osteosarcoma (143B-TK^−^) cell line as previously described [38]. Individual hybrid clones were isolated 10–20 days later using glass cylinders. Hybrid cell lines were cultured as previously described [39]. All reagents for cell culture were from Cambrex-Lonza (Basel, Switzerland) and FBS from Gibco, Invitrogen (Carlsbad, CA, USA).

### Genome sequences

The entire mitochondrial genome was sequenced in a series of overlapping fragments using M13-tagged oligodeoxynucleotide primers to facilitate direct sequencing of the PCR amplified products with BigDye^®^terminator chemistries on an Applied Biosystem 3100 automated sequencer (Applied Biosystems, Warrington, UK) [40]. All sequences were directly compared to the revised Cambridge reference sequence for human mtDNA (GenBank Accession number NC_012920).

### Cell viability assays

#### Trypan blue exclusion assay

2 × 10^5^ cells (2008-C13) or 1 × 10^5^ cells (H2008-HC13) were plated on 6-well plates and, following overnight incubation, were exposed to different treatments according to experimental protocols. After treatments, cells were washed, detached with 0.25% trypsin-0.2% EDTA and suspended in trypan blue (Sigma-Aldrich, St Louis, MO, USA) at 1:1 ratio in medium solution. Cells were counted using a chamber Burker hemocytometer.

#### Sulforhodamine B (SRB) test

2.5 × 10^3^ cells were plated on 96-well plates and, following overnight incubation, were exposed to different treatments according to experimental protocols. After treatments cells were fixed to tissue-culture plates with trichloroacetic acid (Sigma-Aldrich) and stained for 30 minutes with SRB (Sigma-Aldrich). The bound SRB was dissolved by adding 160 μl of 10 mM TRIS (pH = 10.5) and the absorbance was measured at 570 nm using a Victor3X multilabel plate counter (Wallac Instruments, Turku, Finland).

#### Annexin V/propidium iodide staining

1 × 10^5^ cells (H2008-HC13) were seeded in 12-well plates, incubated overnight and treated with cisplatin (1–10 μM) for 24 hours. Then, cells were harvested by quick trypsinization and centrifugated at 1200 rpm for 5 minutes. The cell pellet was resuspended in a binding buffer containing Alexa Fluor 488 Annexin V and propidium iodide (Molecular Probes, Invitrogen, Carlsbad, CA, USA) and then incubated for 15 minutes at room temperature as previously described [12]. The fluorescence of stained cells was measured by Epics XL flow cytometer (Coulter Systems, Fullerton, CA, USA) and analysed with the EXPO 32 software (Coulter Systems, Fullerton, CA, USA).

### Oxygen consumption

Oxygen consumption was measured in live cells (3.5 × 10^6^) resuspended in 1 ml glucose-free DMEM (Gibco, Invitrogen) supplemented with 10% sodium-pyruvate (Cambrex-Lonza) at 37°C, using a Clark-type oxygen electrode (Hansatech Instruments, King's Lynn, Norfolk, UK). Oxygen consumption was measured using the software Oxygraph plus v. 1.01. Data processing includes: fmol/c/min = (RATE/3.5 × 10^6^)*1 million.

### Mitochondrial membrane potential (ΔΨ) and mitochondrial mass

#### Flow cytometry

2 × 10^5^ cells (2008-C13) or 1 × 10^5^ cells (H2008-HC13) were seeded and incubated for 48 hours, washed with phosphate buffer saline solution (PBS), detached with 0.25% trypsin-0.2% EDTA and centrifuged for 5 minutes at 1200 rpm. Cells were resuspended with rhodamine-123 (10 μM) or Acridine Orange 10-Nonyl bromide NAO (25 nM) and incubated for 15 minutes. Probes were from Sigma-Aldrich. Fluorescence intensity was analyzed using an Epics XL flow cytometer. 10^4^ cells of interest were analyzed. Mean fluorescence intensity (MFI) values were obtained using the EXPO 32 software.

#### Live-cells confocal microscopy

2 × 10^5^ cells (2008-C13) or 1 × 10^5^ cells (H2008-HC13) were grown in 3.5 cm glass-bottom dishes (MatTek Corporation, Ashland, USA) and, after 48 hours, were loaded with 100 nM Mitotracker Green (MTG; Invitrogen). Cells were imaged using a laser scanner microscope (Leica TCS SP5, 60X magnification). A volumetric reconstruction was then obtained and analysed using the software Volocity.

### Immunoblot assay

1.5 × 10^6^ cells (2008-C13) were plated in 100 mm cell culture dish and allowed to attach overnight. After 48 hours, cells were lysed with ice-cold lysis buffer supplemented with the protease inhibitor cocktails (Roche Molecular Biochemicals, Mannheim, Germany). The protein content was determined by Lowry procedure (Bio-rad DC Protein Assay, MA, USA). Equal amounts of protein (40 μg) were loaded on a polyacrylamide gel and electrophoretically separated in running buffer. After electrophoresis, the proteins were blotted onto an Hybond-P PVDF membrane (Amersham Biosciences, Buckinghamshire, UK). After blocking, the membrane was exposed to the elected primary antibodies: anti-LC3 (1:1000; Cell Signaling, MA, USA) or anti-G6PD (1:500; Santa Cruz Biotechnology, Inc., Europe). After washing, the membrane was incubated with HRP-conjugated anti-rabbit secondary antibody (1:3500; PerkinElmer, MA, USA). The signal was visualized with enhanced chemoluminescent kit (Amersham Biosciences) according to the manufacturer's instructions and analyzed by Molecular Imager VersaDoc MP 4000 (Bio-rad). LC3 and G6PD were normalized to beta-actin (1:7000; AbCam, Cambridge, UK).

### Quantitative real-time PCR

Total mRNA was isolated as per manufacturer's instructions using QIAshredder and RNeasykits (Qiagen, Venlo, Netherlands) and measured with a NanoDrop ND-1000 spectrophotometer (NanoDrop Technologies, Inc. Wilmington, DE, USA). 0,5 μg of total mRNA was reverse-transcribed to complementary DNA using The High Capacity cDNA Reverse Transcription Kits of Applied Biosystem by Life Technologies. The relative expression of genes of interest was determined by quantitative real-time PCR (StepOne™ Systems of Applied Biosystem by Life Technologies) using Power SYBR^®^ Green PCR Master Mix (Applied Biosystem by Life Technologies) and the primers designed as follow: BNIP3: F gaatttctgaaagttttccttcca R ttgtcagacgccttccaata; GLUT1: F ttaactccacccacctcct, R ccaaatcggcatcttctcat; PFKM: F gccatcagcctttgacaga, R ctccaaaagtgccatcactg; PGK1: F cagctgctgggtctgtcat, R gctggctcggctttaacc; LDHA: F aaaccgtgttattggaagcg, R agcactctcaaccacctgct. To measure the mRNA level of G6PDH and mitochondrial biogenesis genes, total mRNA was isolated with TRIzol (Life Technologies) as previously described by Chomczynski P and Sacchi N [42] and measured with a Beckman Coulter DU-800 spectrophotometer. The relative expression of each gene was determined by quantitative real-time PCR (Eco™ Illumina, Real-Time PCR system, San Diego, CA, USA) using One Step SYBR PrimeScript RT-PCR Kit (Takara Bio, Inc., Otsu, Shiga, Japan) and the primers designed as follow: G6PDH: F aagaacgtgaagctccctga R aatataggggatgggcttgg; PGC-1α: F acacagtcgcagtcacaacac R ggagtggtgggtggagttagg; PGC-1β: F gcacctcacctcggcacag R cggctccttgtcctccttgg; NRF1: F gtaaccctgatggcactgtctc R gcttgcgtcgtctggatgg; NRF2: F ttccttcagcagcatcctctcc R aatctgtgttgactgtggcatctg; Tfam: F aacaacgaaaatatggtgctgagg R caagtattatgctggcagaagtcc. Linearity and efficiency of PCR amplifications were assessed using standard curves generated by serial dilution of complementary DNA; melt-curve analysis was used to confirm the specificity of amplification and absence of primer dimers. All genes were normalized to β-actin designed as follow: F ccaaccgcgagaagatga R ccagaggcgtacagggatag. Expression levels of the indicated genes were calculated by the ΔΔCt method using respectively the dedicated StepOne software or Eco™ Software v4.0.7.0.

### Liquid chromatography-mass spectroscopy (LC-MS)

2 × 10^5^ cells (2008-C13) were plated in 6-well plates and after 48 hours quickly washed with ice cold PBS on an ice bath. The samples were therefore prepared as previously described [43]. In brief, cells were lysed with a dry ice/methanol solution (−80°C) of 50% methanol/30% acetonitrile in water and quickly scraped. The extracts were mixed at 4°C for 15 minutes and pelleted in a cooled centrifuge (4°C). The supernatant was collected and submitted for LC-MS analysis. The amount of extraction solution was calculated according to the number of cells present in the sample dish, extrapolated using a “counter dish” cultured in the same conditions of the sample dishes. A concentration of 1 ml per 1 × 10^6^ cells was used in the extraction solutions.

Intermediates were separated using a liquid chromatography system. Data acquisition was controlled with Xcalibur 2.0 (ThermoElectron Co, San Jose, CA, *USA*). The mass accuracy was maintained below 1 ppm due to use of a lock mass. The raw chromatograms were then aligned using the software SIEVE™ (ThermoElectron). The integration of the measured ion current over a metabolite's elution time and *m/z* interval is directly proportional to its absolute abundance in the solution. We manually removed from the SIEVE's output unspecific and misaligned peaks to eliminate the noise.

### Glucose uptake

5 × 10^3^ cells (2008-C13) were plated in 96-well plate and allowed to attach overnight. After 24 hours, glucose uptake was measured by incubating cells with 90 μM glucose analogue 6-NBDG (Invitrogen, Paisley, UK) for 1 minute. Cells where then washed, added with PBS and their fluorescence (λex: 465 nm, λem: 540nm) was measured by Victor3X multilabel plate counter (Wallac Instruments).

### GSH/GSSG

Cellular GSH and GSSG content was enzymatically determined as previously described by Floreani *et al*. [44]. 1.5 × 10^6^ cells (2008-C13) were plated in 100 mm cell culture dish and allowed to attach overnight. After 48 hours, cells were washed with PBS and then treated with 6% meta-phosphoric acid (MPA). After 10 minutes at room temperature, the acid extract was collected, centrifuged for 5 minutes at 14000 rpm and processed. The cellular pellet was solubilized with 0.5 M potassium hydroxide and assayed for protein content. For total GSH determination, the above acid extract was diluted in 6% MPA and added with 0.1 M potassium phosphate/5 mM EDTA buffer (pH 7.4), 10 mM 5,5-dithiobis-(2-nitrobenzoic acid) and 5 mM NADPH. After a 3 minute equilibration period, the reaction was started by adding 2 U GSH reductase (type III; Sigma-Aldrich; from baker's yeast; diluted in 0.1 M phosphate/EDTA buffer). Product formation was recorded continuously at 412 nm with a Beckman DU800 UV-Vis spectrophotometer. The total amount of GSH was determined from a standard curve obtained by plotting known amounts of GSH against the rate of change in absorbance. For GSSG measurement, soon after preparation, the supernatant of acid extract was treated for derivatization with 2-vinylpyridine and triethanolamine. The samples were incubated at room temperature for 60 minutes and then assayed with the same procedure above described for total GSH measurement. The GSH concentration of each sample was calculated as the difference between total glutathione and GSSG. All reagents were from Sigma-Aldrich.

### G6PDH activity

1.5 × 10^6^ cells (2008-C13) were plated in 100 mm cell culture dish and allowed to attach overnight. After 48 hours, cells were washed with PBS and quickly scraped. 2.5 × 10^6^ of cells were then collected by centrifugation and sonicated on ice. The G6PDH activity was assayed on cell supernatant as per manufacturer's instructions using the Glucose-6-Phosphate Dehydrogenase Activity Assay Kit (Cayman Chemical Company, MI, USA). The fluorescence intensity (λex/em = 540/585) was measured using a Victor3X multilabel plate counter (Wallac Instruments). The G6PDH activity (nmol/min/ml) was calculated as per manufacturer's instructions.

### Statistical analyses

All data are expressed as mean ± SEM. Standard ANOVA procedures followed by multiple pairwise comparison adjusted with Bonferroni corrections were performed for cell viability assays. Unpaired Student's *t*-tests were used to analyse all the other results. Significance was considered at *p* < 0.05.

#### Isobolographic analysis

Isobolographic analysis was used to determine the effect of the combination between cisplatin and the G6PDH inhibitor 6-AN or DHEA. Isoboles are defined as iso-effect curves that show drug concentrations resulting in equal effect [45, 46]. From iso-effective curves it is possible to verify the presence of simple additivity, supra-additivity (synergism, i.e. when drug combination produces an effect greater than that predictable from each drug alone) or infra-additivity (antagonism). Isobolographic analysis was possible only for C13 and A431pt data; in 2008 cells the predominant effect produced by cisplatin hindered the overall effect.

## SUPPLEMENTARY MATERIALS AND METHODS FIGURES AND TABLE


