# Supplementary material for: Inhibition of glucose-6-phosphate dehydrogenase sensitizes cisplatin-resistant cells to death
Source: Oncotarget. 2015 Aug 17;6(30):30102–14. doi: 10.18632/oncotarget.4945 (PMC4745784; doi:10.18632/oncotarget.4945)
Supplement: Supplementary file 1 [file oncotarget-06-30102-s001.pdf]

SUPPLEMENTARY MATERIALS AND METHODS

CELL LINES

Human cervix squamous cell lines

(A431 wild type and A431pt cisplatin resistant cells) were grown in Roswell Park Memorial Institute medium (RPMI 1640) supplemented with 10% fetal bovine serum (FBS), 4 mM glutamine, 100 U/ml penicillin and 100 μg/ml streptomycin, in humidified condition at 5% CO_2 and 37°C. All reagents for cell culture were from Cambrex-Lonza (NY, USA) and FBS from Gibco, Invitrogen (Carlsbad, CA, USA).

CELL VIABILITY ASSAYS

Trypan blue exclusion assay

1.5×10^5 cells (2008-C13) or 7.5×10^4 cells (A431-A431pt) were plated on 12-well plates and, following overnight incubation, were exposed to different treatments according to experimental protocols. After treatments, cells were washed, detached with 0.25% trypsin-0.2% EDTA and suspended in trypan blue (Sigma-Aldrich, St Louis, MO, USA) at 1:1 ratio in medium solution. Cells were counted using a chamber Burker hemocytometer.

Sulforhodamine B (SRB) test

2.5×10^3 cells were plated on 96-well plates and, following overnight incubation, were exposed to different treatments according to experimental protocols. After treatments cells were fixed to tissue-culture plates with trichloroacetic acid (Sigma-Aldrich) and stained for 30 minutes with SRB (Sigma-Aldrich). The bound SRB was dissolved by adding 160 μl of 10 mM TRIS (pH = 10.5) and the absorbance was measured at 570 nm using a Victor3X multilabel plate counter (Wallac Instruments).

GLUCOSE UPTAKE

5×10^3 cells (A431-A431pt) were plated in 96-well plate and allowed to attach overnight. After 24 hours, glucose uptake was measured by incubating cells with 90 μM glucose analogue 6-NBDG (Invitrogen, Paisley, UK) for 10 minute. Cells were then washed, added with PBS and their fluorescence (λex: 465 nm, λem: 540nm) was measured by Victor3X multilabel plate counter (Wallac Instruments).

IMMUNOBLOT ASSAY

1×10^6 cells (A431-A431pt) were plated in 100 mm cell culture dish and allowed to attach overnight. After 48 hours, cells were lysed with ice-cold lysis buffer supplemented with the protease inhibitor cocktails (Roche Molecular Biochemicals). The protein content was determined by Lowry procedure (Bio-rad DC Protein Assay). Equal amounts of protein (40 μg) were loaded on a polyacrylamide gel and electrophoretically separated in running buffer. After electrophoresis, the proteins were blotted onto an Hybond-P PVDF membrane (Amersham Biosciences). After blocking, the membrane was exposed to the elected primary antibodies: anti-GLUT1 (1:1000; Abcam) or anti-G6PD (1:500; Santa Cruz Biotechnology, Inc.). After washing, the membrane was incubated with HRP-conjugated anti-rabbit secondary antibody (1:3500; PerkinElmer). The signal was visualized with enhanced chemoluminescent kit (Amersham Biosciences) according to the manufacturer's instructions and analyzed by Molecular Imager VersaDoc MP 4000 (Bio-rad). GLUT1 and G6PD were normalized to GAPDH (1:2000; Santa Cruz Biotechnology, Inc.).

QUANTITATIVE REAL-TIME PCR

To measure the mRNA level of G6PD, total mRNA was isolated with TRIzol (Life Technologies) as previously described by Chomczynski P and Sacchi N [42] and measured with a Beckman Coulter DU-800 spectrophotometer. The relative expression of each gene was determine by quantitative real-time PCR (Eco™ Illumina, Real-Time PCR system) using One Step SYBR PrimeScript RT-PCR Kit (Takara Bio, Inc.) and the primers designed as follow: G6PDH: F aagaacgtgaagctccctga R aatataggggatgggcttgg. Linearity and efficiency of PCR amplifications were assessed using standard curves generated by serial dilution of complementary DNA; melt-curve analysis was used to confirm the specificity of amplification and absence of primer dimers. G6PD was normalized to GAPDH designed as follow: F ctgacttcaacagcgacacc R gtggtccaggggtcttactc. Expression levels of the indicated genes were calculated by the ΔΔCt method using respectively the dedicated StepOne software or Eco™ Software v4.0.7.0.

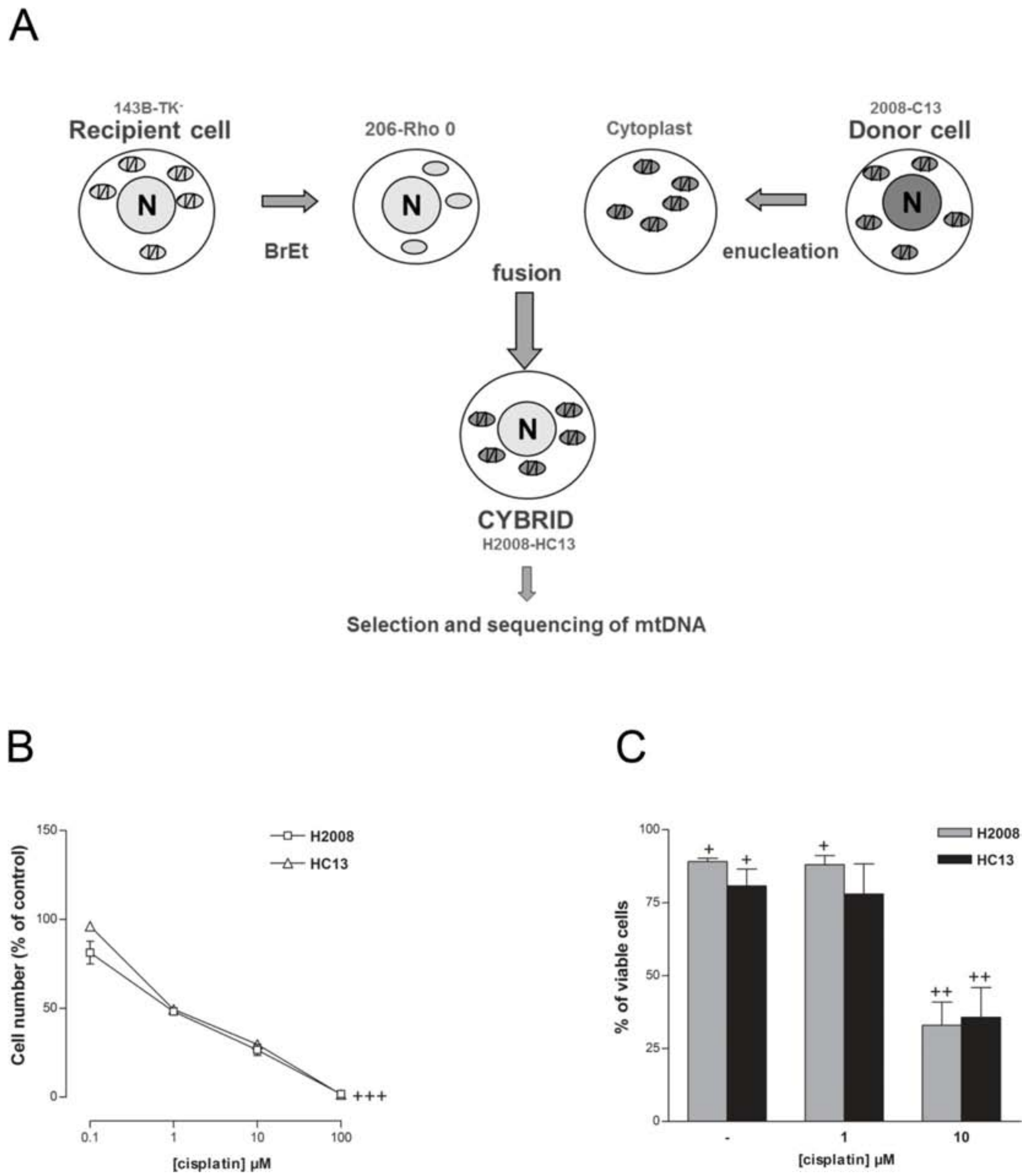

Supplementary Figure S1: Transmitochondrial hybrid cell model. A. Schematic representation of cybrids (H2008-HC13) generation. **B-C.** Effect of cisplatin on cybrid cell viability measured by trypan blue exclusion assay (B) and by flow cytometry (C) The data were obtained from at least three independent cultures. +++$p < 0.001$, ++$p < 0.01$, +$p < 0.05$; treatment *vs* control.

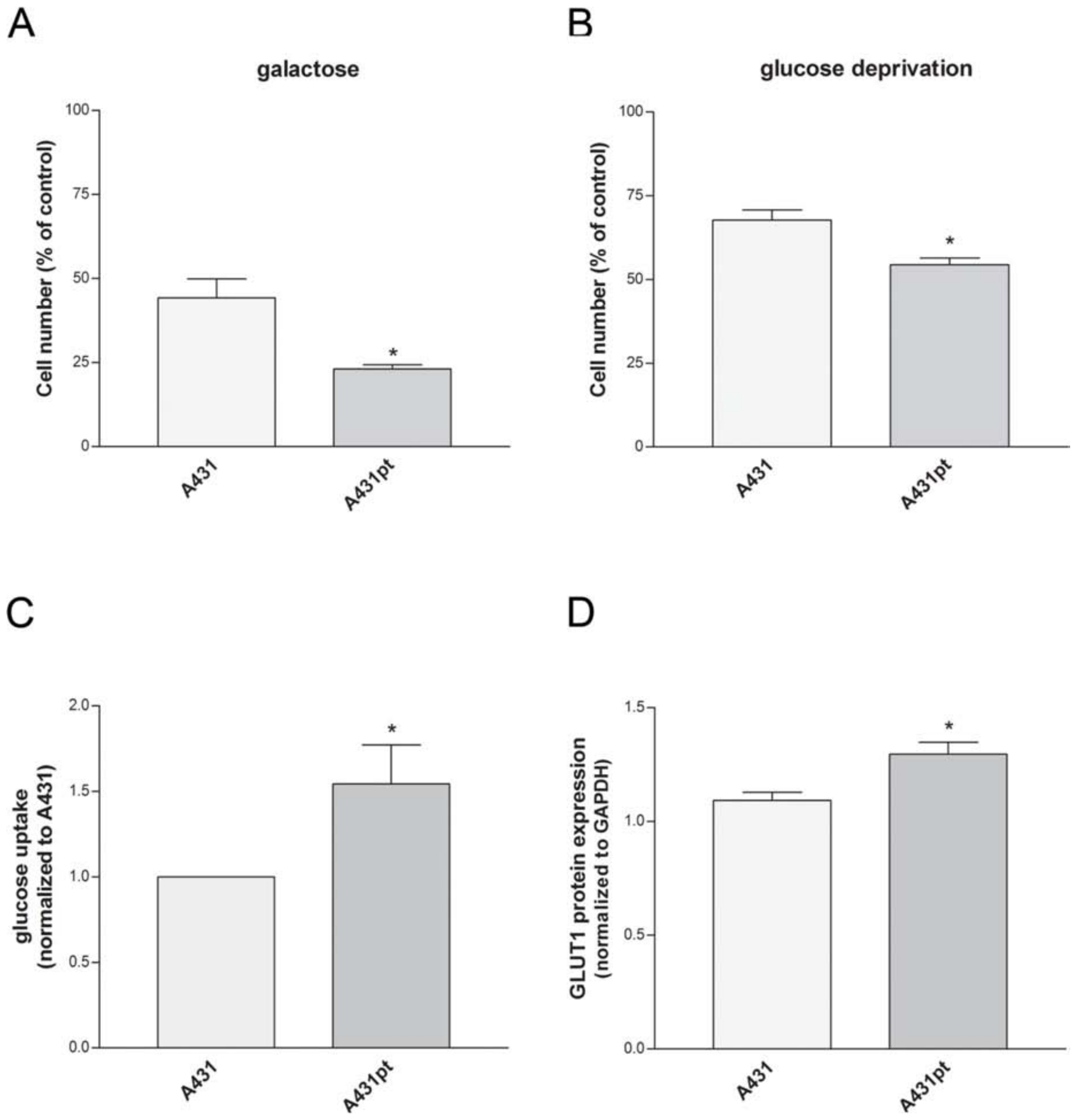

Supplementary Figure S2: Cisplatin-resistant cells show an increased dependency to glucose. A. Effect of 5 mM galactose and **B.** glucose deprivation on cell viability after 24 hours of treatment in A431 and A431pt cell lines. Data are expressed as percentage of cell number compared to the relative control. **C.** Glucose uptake measured after incubation with the glucose analogue 6-NBDG. Data are normalized to cisplatin-sensitive cells. **D.** Protein expression GLUT1 measured by western blotting. GLUT1 was normalized to GAPDH. The data were obtained from at least 3–4 independent cultures. *$p < 0.05$; A431pt *vs* A431.

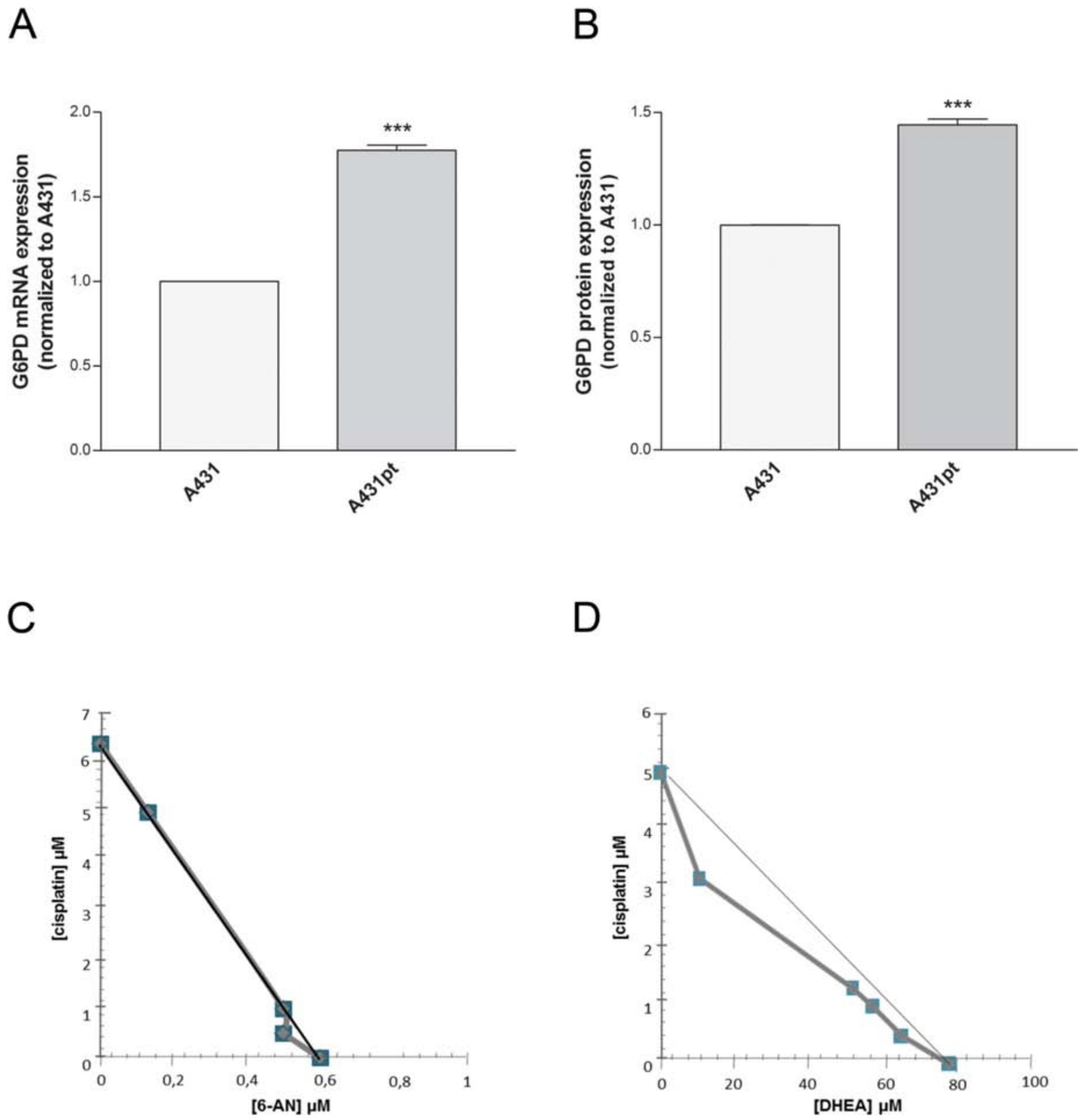

Supplementary Figure S3: Inhibition of G6DP, sensitizes cisplatin-resistant cells. A–B. mRNA levels and protein expression of G6PD. Data are expressed as ratio of cisplatin-resistant cell line (A431pt) on the sensitive counterpart (A431). **C–D.** Isobologram showing the effect of 6AN or DHEA administered in association with cisplatin treatment on cisplatin-resistant A431pt cells. Data are expressed as percentage of cell number compared to control. The graph was obtained using iso-effective drug concentrations causing 25% of cytotoxic effect. Straight line indicates the theoretical additivity line. The data were obtained from at least 3–5 independent cultures. ****p* < 0.001; A431 *vs* A431pt.

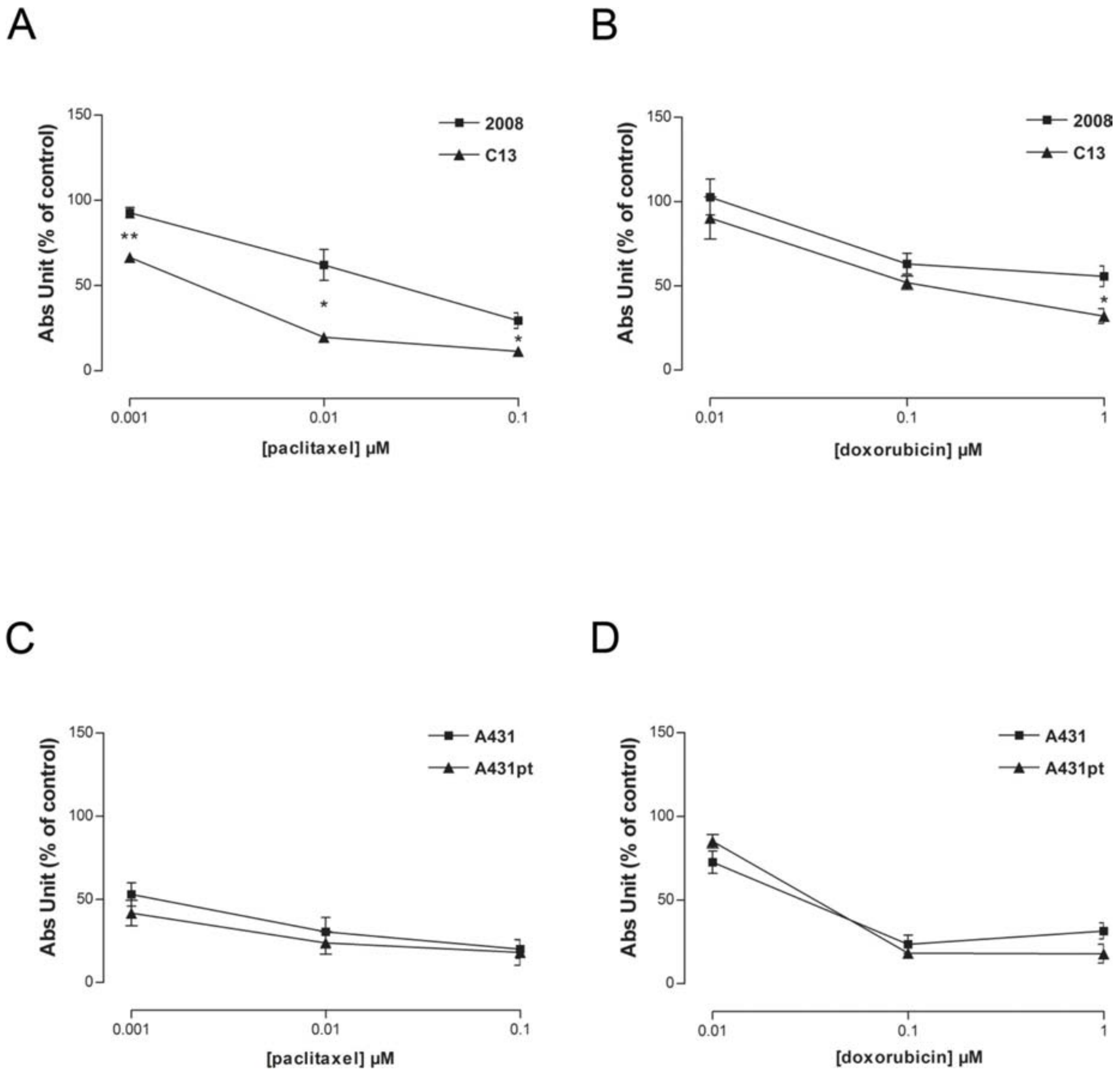

Supplementary Figure S4: Cisplatin-resistant cells do not display cross-resistance. Effect of paclitaxel (0.001–0.1 μM) and doxorubicin (0.01–1 μM) on cell viability after 24 hours of treatment. Data are expressed as percentage of cell number compared to the relative control. The data were obtained from at least 3–4 independent cultures. $^{**}p < 0.01$, $^{*}p < 0.05$; C13 *vs* 2008.

Supplementary Table S1: GSH *vs* GSSG ratio

	GSH	GSSG	GSH/GSSG
**2008**	28,07 ± 3,76	0,34 ± 0,09	**85,54** ± 15,11
**C13**	63,20 ± 6,83	0,67 ± 0,13	**96,73** ± 17,4
